# Stress Concentration of Endodontically Treated Molars Restored with Transfixed Glass Fiber Post: 3D-Finite Element Analysis

**DOI:** 10.3390/ma14154249

**Published:** 2021-07-29

**Authors:** Alexandre Luiz Souto Borges, Manassés Tercio Vieira Grangeiro, Guilherme Schmitt de Andrade, Renata Marques de Melo, Kusai Baroudi, Laís Regiane Silva-Concilio, João Paulo Mendes Tribst

**Affiliations:** 1 Department of Dental Materials and Prosthodontics, Institute of Science and Technology, São Paulo State University (Unesp), São José dos Campos, São Paulo 12245-000, Brazil; alexandre.borges@unesp.br (A.L.S.B.); manasses.grangeiro@unesp.br (M.T.V.G.); guisdandrade@hotmail.com (G.S.d.A.); renata.marinho@unesp.br (R.M.d.M.); 2 Graduate Program in Dentistry, Department of Dentistry, University of Taubaté (UNITAU), Taubaté 12020-270, Brazil; d_kusai@yahoo.co.uk (K.B.); regiane1@yahoo.com (L.R.S.-C.)

**Keywords:** dental restoration failure, endodontically treated teeth, finite element analysis, dental materials

## Abstract

The loss of dental structure caused by endodontic treatment is responsible for a decrease in tooth resistance, which increases susceptibility to fracture. Therefore, it is important that minimally invasive treatments be performed to preserve the dental structure and increase the resistance to fracture of endodontically treated posterior teeth. To evaluate under axial loads, using the finite element method, the stress distribution in endodontically treated molars restored with both transfixed or vertical glass fiber posts (GFP) and resin composite. An endodontically treated molar 3D-model was analyzed using finite element analyses under four different conditions, class II resin composite (G1, control model), vertical glass fiber post (G2), transfixed glass fiber posts (G3) and vertical and transfixed glass fiber posts (G4). Ideal contacts were considered between restoration/resin composite and resin composite/tooth. An axial load (300 N) was applied to the occlusal surface. The resulting tensile stresses were calculated for the enamel and dentin tissue from five different viewports (occlusal, buccal, palatal, mesial and distal views). According to the stress maps, similar stress trends were observed, regardless of the glass fiber post treatment. In addition, for the G1 model (without GFP), a high-stress magnitude can be noticed in the proximal faces of enamel (7.7 to 14 MPa) and dentin (2.1 to 3.3 MPa) tissue. The use of transfixed glass fiber post is not indicated to reduce the stresses, under axial loads, in both enamel and dentin tissue in endodontically treated molar with a class II cavity.

## 1. Introduction

The loss of dental structure caused by endodontic treatment is responsible for a decrease in tooth resistance, which increases susceptibility to fracture [1]. The longevity of endodontically treated teeth is influenced by several factors, such as, the preservation of remaining dental tissue, effectiveness of restorative procedures and occlusal force [2]. Therefore, it is extremely important that minimally invasive dental treatments be performed to preserve the dental structure and obtain success. Additionally, the literature reports that different types of restoration parameters can increase the resistance to fracture of endodontically treated posterior teeth [3].

One of the most common post-endodontic treatments is glass fiber post (GFP) associated with adhesively bonded resin composite restoration in order to increase fracture resistance and reduce the interfacial gap between dental tissues and restorative materials [4,5]. Some authors justify their use because GFP can distribute chewing stresses and occlusal loads on the restoration [6].

In addition, the elastic modulus of post and the direct restorative material must be compatible with the root dentin to reduce the possibility of fracture [7], as well as the root stress magnitude during chewing loads. Aiming to improve the beneficial effects of GFP usage in weakened teeth, several studies have investigated how different clinical parameters can modify the mechanical response during loading, such as GFP geometries, relining, position, and length. Furthermore, previous in vitro studies reported that inserting transfixed GFP could be a viable alternative procedure to reinforce the coronal dental structure, replacing metallic or ceramic posts [8,9]. According to a clinical study, this procedure is also economically viable and preserves the natural tooth structure compared to full crown preparation [10].

In this sense, several studies aimed to evaluate the restorative techniques that could reinforce the remaining dental structure, to reduce the stress concentration in the dental structure [11,12] and the probability of fracture through alternative restorative procedures [13]. One of these proposed techniques is the use of transfixed GFP in the tooth crown, and is reported as a contemporary conservative treatment [10]. According to the literature, this restorative treatment has satisfactory aesthetics and easy execution compared to full-crown preparations [14]. However, the mechanical effect of transfixed post placement has not yet been extensively investigated in the literature. In vitro studies are controversial about the mechanical improvements in the fracture load when transfixed glass fiber posts were used to restore posterior teeth [8,9,15]. However, no study has evaluated how the transfixed GFP placement can modify the tooth biomechanical behavior and how the stress can be reduced during compressive loading. Therefore, the aim of the present study was to evaluate the stress distribution in endodontically treated molars restored with both transfixed or vertical glass fiber posts and resin composite under axial loads using 3D finite element analysis (3D-FEA). The use of 3D-FEA is the most widely used numerical method, allowing the reproduction of mechanical behavior under a mechanical load based on the properties of the materials [16,17,18,19,20,21].

## 2. Materials and Methods

### 2.1. Modelling

**Figure 1:**
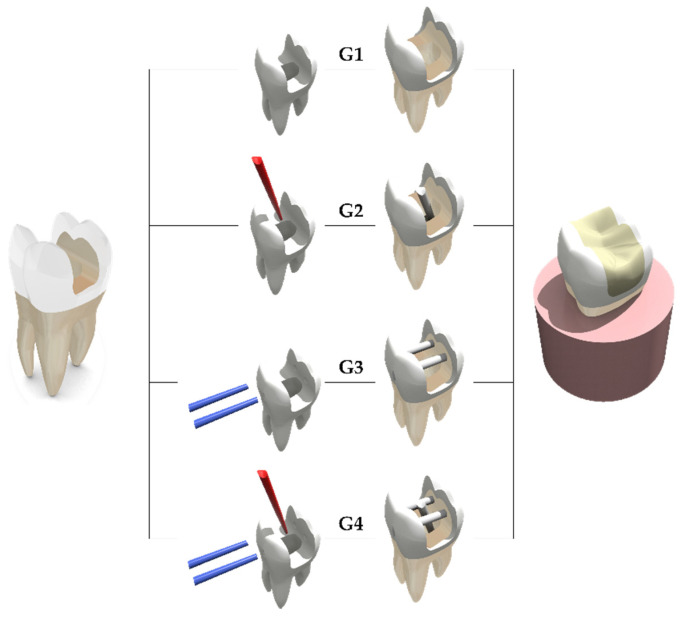
Three-dimensional models created in the modeling software with different post-endodontic restorative treatments: (**G1**) no-post approach; (**G2**) glass fiber cemented in the palatal root canal; (**G3**) two transfixed glass fiber posts; and (**G4**) two transfixed glass fiber posts and one glass fiber post in the palatal root canal. In red the conventional glass fiber post and in blue the transfixed glass fiber posts.

Finite Element Analysis (FEA) was used to evaluate the mechanical behavior and stress distribution in mesio-occluso-distal class II direct resin composite restoration of maxillary first molar, restored endodontically, and treated with four post-endodontic restorative treatments, including a no-post approach (G1, no-post approach), glass fiber cemented in the palatal root canal (G2), two transfixed glass fiber posts (G3), two transfixed glass fiber posts, and one glass fiber post in the palatal root canal (G4) (Figure 1).

**Figure 2:**
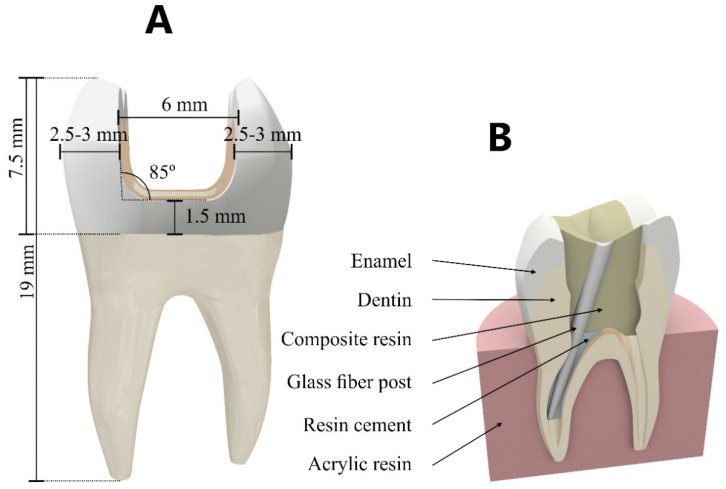
CAD modeling. (**A**) Class II cavity design; (**B**) model components.

The three-dimensional models were designed in NURBS (non-uniform rational basis spline) CAD (computer-aided design) software (Rhinoceros 6.0SR8, McNell North America, Seattle, WA, USA). An intact first upper molar tooth model (previously reported [16], consisting of enamel layer, dentin layer, root, and pulp chamber) was used to generate the evaluated models. Endodontic treatment was designed using the crown-down technique with 4% conicity and 25% tapering. A large class II mesio-occluso-distal (MOD) cavity was performed with 2 ± 0.5 mm thickness of remaining wall, 6 mm isthmus preparation, and gingival wall of 1.5 mm from the cementoenamel junction (Figure 2). An acrylic resin cylinder was designed to simulate the fixation support, in which the models were positioned, exposing 2 mm below the restoration margin. The resulting model was used to simulate the evaluated treatments.

### 2.2. Pre-Processing

**Figure 3:**
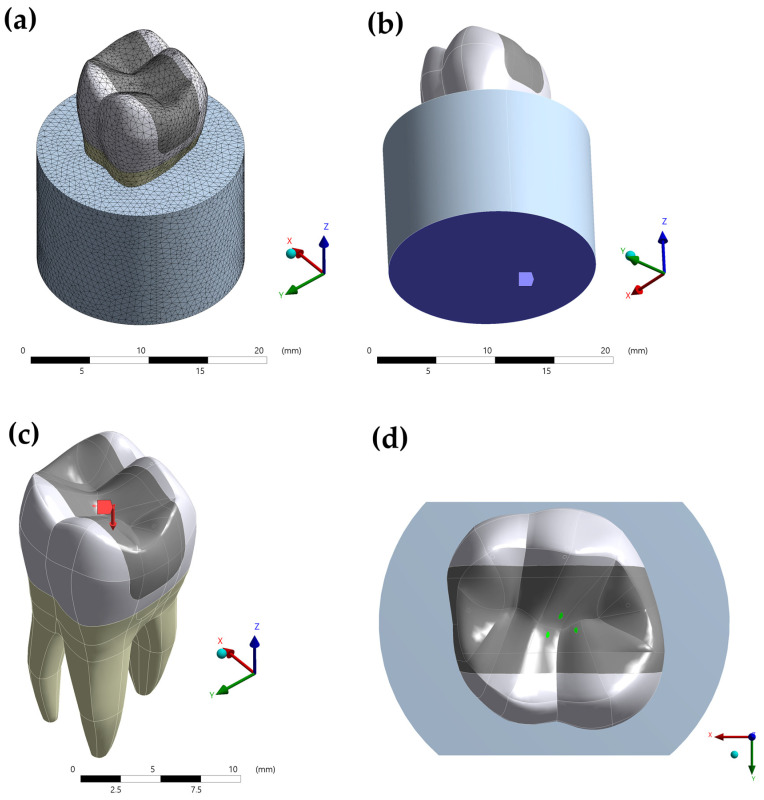
Boundary condition in the present simulation. (**a**) Mesh generation; (**b**) fixation of the system; (**c**) loading settings; (**d**) tripod contact area.

The models were imported to a computer-aided engineering (CAE) software program (ANSYS 19.2, ANSYS Inc., Houston, TX, USA) as a standard for the exchange of product model data (STP) file. Structural mechanical analysis was applied to each group and all materials were considered homogeneous, elastic, and isotropic, except for the glass fiber post, which was considered orthotropic. The interfaces in all models were considered perfectly bonded. Table 1 [20,22,23,24,25,26,27,28,29] summarizes the mechanical properties (elastic modulus and Poisson ratio) used for the mechanical analysis. An average of 156,252 nodes and 128,242 tetrahedral, ten nodes, elements were used for the meshing process after a convergence test with a 10% degree of freedom of the converged value and mesh size, based on the maximum principal stress (MPS) results. During the boundary conditions, the models were fixed (3-axis) on the bottom surface of a cylinder and loaded with 300 N (90°) distributed in tripod contact area at the central fossa (Figure 3).

The stress distribution in enamel and dentin was recorded as colorimetric maps with adjustable color scales corresponding to the stress magnitude comparison among the preparation designs for each analyzed structure.

## 3. Results

**Figure 4:**
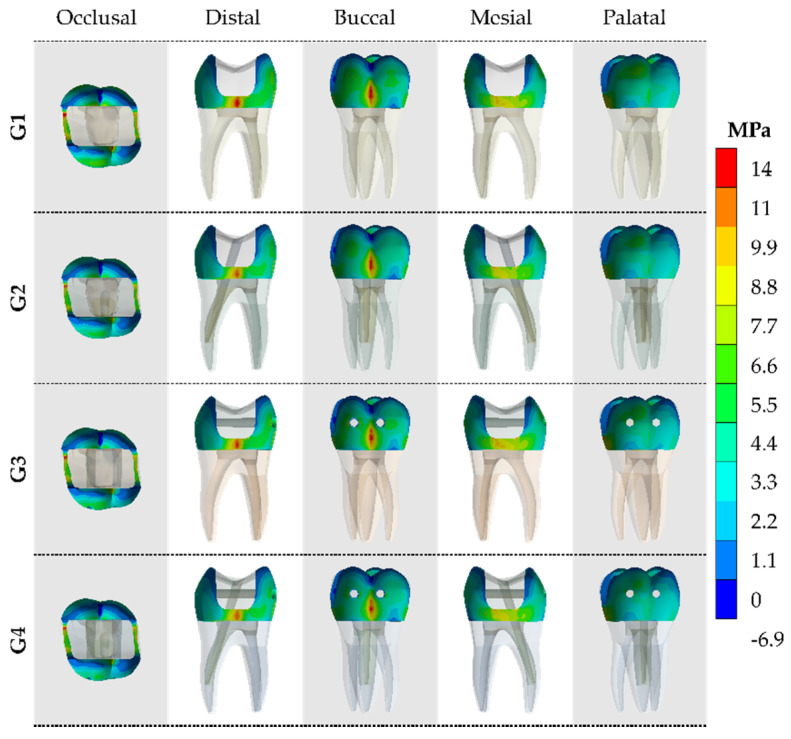
The maximum principal stress (MPa) concentration from five different viewpoints in the enamel tissue according to the GFP treatment.

**Figure 5:**
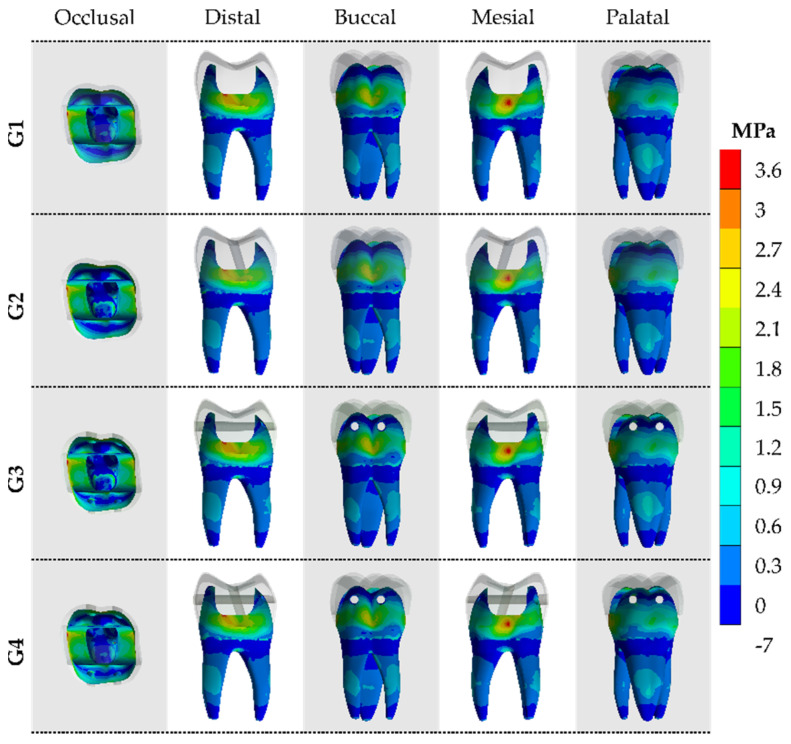
The maximum principal stress (MPa) concentration from five different viewpoints in the dentin tissue according to the GFP treatment.

**Figure 6:**
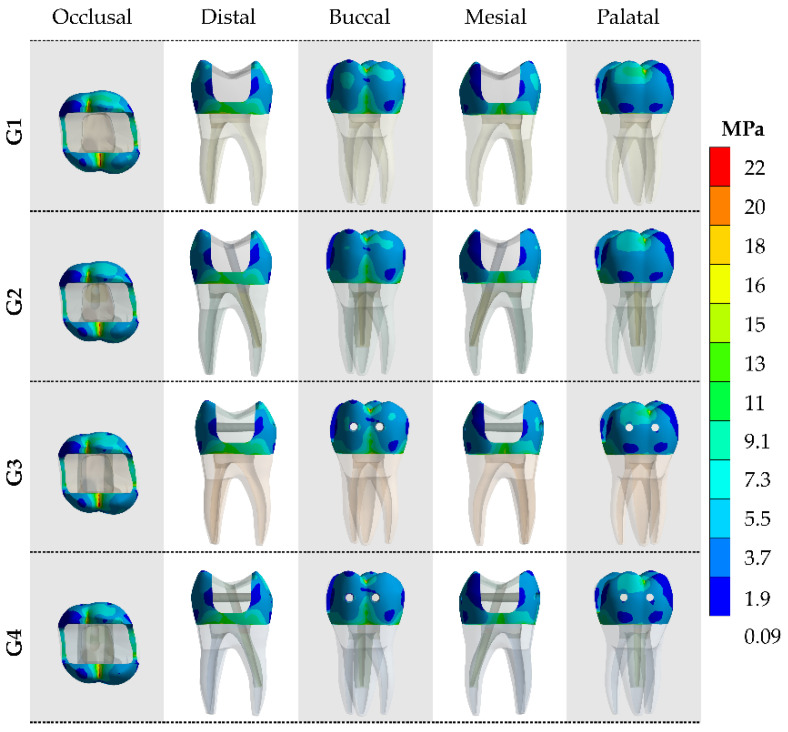
The von-Mises stress (MPa) concentration from five different viewpoints in the enamel tissue according to the GFP treatment.

**Figure 7:**
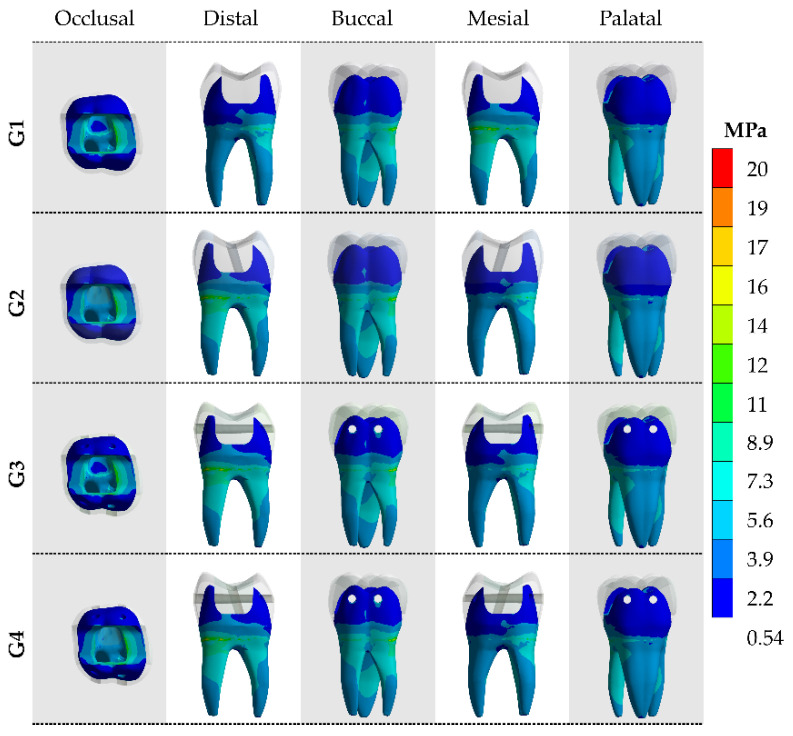
The von-Mises stress (MPa) concentration from five different viewpoints in the dentin tissue according to the GFP treatment.

After the processing of first principal stress (tensile), the results were calculated for the models in each of the tooth faces (occlusal, buccal, mesial, palatal and distal). The tensile (Figure 4 and Figure 5) and von-Mises (Figure 6 and Figure 7) stress data were summarized using stress maps for the enamel tissue and dentin tissue. According to the qualitative results, a similar stress trend was observed regardless of the glass fiber post treatment. In addition, for the G1 model (without GFP), a high stress magnitude can be noticed with more presence of red and yellow fringes in the proximal faces of enamel and dentin tissue. To quantify the model’s comparison, the stress peaks were recorded and are summarized in Table 2 and Table 3.

## 4. Discussion

First molars are teeth frequently involved in endodontic therapy [27]; therefore, the use of resin composite restorations after endodontic treatments should improve the mechanical resistance against the occlusal loads, as well as the restoration of missing dental tissue. However, this mechanical effect is not a consensus in the literature. The failures in endodontically treated teeth are still widely reported even after restorative procedures.

Previous reports showed that the fracture resistance during in vitro compressive loads could be enhanced with the GFP transfixed placement in posterior teeth [8,14,30]. There are reports affirming that the use of two transfixed GFPs in MOD-prepared cavities led to recovery of approximately 23% more fracture strength than teeth without GFPs [30]. According to the authors, a possible explanation would be the reduction of cusp deflection caused by anchoring of buccal and lingual walls of the cavity. The present study showed a slight stress reduction with the use of GFP regardless of the post-endodontic treatment that could be associated with the reduction in cusp’s displacements; however, with values lower than 2% in enamel tissue and 8% in dentin tissue comparing G1 and G3. Therefore, it is possible to suggest that the GFP effect would be more noticeable when the adhesion is not ideal between resin composite restoration and cavity walls.

The mechanical properties of the restorative materials can determine the clinical performance of restored endodontically treated teeth, especially the elastic modulus of the post system [31]. Rigid posts, such as metallic and zirconia, generates less stress in the cement layer, however, concentrate more on root dentin and, thus, catastrophic root fractures can occur if the tooth is overloaded. On the other hand, less rigid posts, like the fiber-reinforced posts, can deflect under high loads, which can lead to loss of retention, or even post fracture, however, avoiding root fracture [32]. In this regard, the present study is limited to the mechanical behavior with the use of GFP, however different post systems or transfixed reinforcement systems may modify the calculated stress and should be evaluated in further evaluations.

Another in vitro study [33] reported that the transfixed glass fiber post placement could be an alternative treatment modality for the restoration of endodontically treated teeth. According to the authors, this technique did not improve the fracture resistance of endodontically treated teeth with MOD cavities; the present study corroborates this, since the difference in stress magnitude between models is less than 10%. According to the authors, this mechanical behavior can be explained due to the minimal surface area between the GFP and the tooth structure; hence, it does not provide an adequate area for bonding. In addition, the GFP bond strength with resin cement is weaker than the bond strength between the composite restoration and dental tissue. Finally, the presence of holes in the crown might have affected the fracture resistance of teeth. Therefore, the present study suggests that the elastic modulus of GFP is lower than the enamel, and hence would present considerable flexible structure that cannot act as a stress reduction framework in this case.

Cusp deflection mainly occurs along the bucco-lingual axis and usually occurs because most of the chewing forces on posterior teeth are directed laterally [15]. These directional oblique components of the masticatory load can affect the adhesive layer in MOD cavities, being mandatory the use of effective adhesive systems to retain the restorative materials. In addition to that, with the interface property, the enamel tissue can be considered a brittle material, while dentine is more elastic damping the stress effect at the dentin-enamel junction [15].

On the other hand, when associated with the insertion of GFP in the palatal root canal (G2), there is slightly improvement in the absorption of occlusal loads, associated with stress dissipation along its axis [34], resulting in an improvement in the tooth resistance [35]. This effect has been observed in clinical reports [36,37] corroborating with the results found in the present study.

Another finite element study [38] showed that the use of adhesive GFP was neither able to reduce the maximum stresses calculated on the occlusal surface nor to optimize the stress distribution regardless of different vertical post-approach. According to the authors, the placement of high amount of GFP can be deleterious to the remaining tooth structure without improving the mechanical response against chewing loading. The present study corroborates with this indication, since there use of more GFPs (G4) was not beneficial for the present model stress result.

Although in vitro laboratory tests of extracted human teeth are important to obtain useful information about fracture load and the fracture mode, they are generally based on destructive experiments and have limited capacity to investigate the stress-strain relationship in the tooth restoration complex [38,39]. Therefore, 3D-FEA is an engineering tool that can be applied to biology, medicine and dentistry and used to investigate the mechanical behavior of complex systems by a mathematical approach and simulation [37]. The numerical simulation consists of modeling a structure as close as possible to the real one, in addition to the correct outputs that should be applicable and practical based in the clinical research to define the boundary conditions. However, restorations have other problems, such as microleakage, polymerization shrinkage of resinous materials and postoperative sensitivity, that should be considered. As oral conditions cannot be completely reproduced by in vitro and in silico studies, further evaluations are still necessary to determine the effectiveness and longevity of GFP treatment in class II MOD cavities [40].

In addition, based on what was said before, the FEA presents limitations related to numerical simulation. Initially, the endodontically treated teeth would be subject to different temperature cycles and pH variation in the oral cavity. In addition, the simulated materials would present some defects that are not simulated in isotropic structures [41]. There are, possible influences of oblique loading, sliding contacts and operator errors that are simplified [42]. Therefore, further in vitro studies should be carried out to complement the present findings, demonstrating transfixed GFP biological behavior, fatigue survival, and bond strength followed by clinical trials.

## 5. Conclusions

Based on this study’s limitations, the use of transfixed glass fiber post generated stresses similar to the absence of a post and is not indicated to improve the endodontically treated molar mechanical response, in both enamel and dentin tissue. The conventional glass fiber post placement is the most suitable technique to reduce the stress magnitude during axial loading.

## Figures and Tables

**Table 1:** Stress peaks (MPa) obtained in enamel and dentin tissue after the analysis process.

Material	Elastic Modulus (GPa)	Shear Modulus (GPa)	Poisson Ratio	Tensile Strength (MPa)
enamel	84 [23]	-	0.3 [23]	15.1–34.3 [28]
dentin	18.6 [24]	-	0.3 [24]	44.4–97.8 [29]
resin composite	8.0 [25]	-	0.25 [25]	-
glass fiber post	x = 37 [26] y = 9.5 [26] z = 9.5 [26]	xy = 3.1 [20] xz = 3.5 [20] yz = 3.1 [20]	xy = 0.27 [26] xz = 0.34 [26] yz = 0.27 [26]	-
resin cement	7 [25]	-	0.24 [25]	-
acrylic resin	2.2 [25]	-	0.3 [25]	-

**Table 2:** Tensile stress peaks (MPa) obtained in enamel and dentin tissue after the analysis process.

Model	Glass Fiber Post Approach	Enamel Stress	Dentin Stress
G1	No-post	14.5	3.7
G2	Glass fiber in the palatal root canal	13.9	3.2
G3	Two transfixed glass fiber posts	14.2	3.4
G4	Two transfixed glass fiber posts and one glass fiber post in the palatal root canal.	14.0	3.3

**Table 3:** Von-Mises stress peaks (MPa) obtained in enamel and dentin tissue after the analysis process.

Model	Glass Fiber Post Approach	Enamel Stress	Dentin Stress
G1	No-post	18.17	11.68
G2	Glass fiber in the palatal root canal	17.58	11.18
G3	Two transfixed glass fiber posts	17.87	11.38
G4	Two transfixed glass fiber posts and one glass fiber post in the palatal root canal.	17.68	11.28

## Data Availability

The data presented in this study are available on request from the corresponding author.
